# GPS tracking for mapping seabird mortality induced by light pollution

**DOI:** 10.1038/srep10670

**Published:** 2015-06-02

**Authors:** Airam Rodríguez, Beneharo Rodríguez, Juan J. Negro

**Affiliations:** 1 Department of Evolutionary Ecology, Estación Biológica de Doñana (CSIC), Avda. Américo Vespucio s/n, 41092 Seville, Spain; 2 Research Department, Phillip Island Nature Parks, P.O. BOX 97, 3922 Cowes, Victoria, Australia; 3 Canary Islands’ Ornithology and Natural History Group (GOHNIC), La Malecita s/n, 38480 Buenavista del Norte, Tenerife, Canary Islands, Spain

## Abstract

Light pollution and its consequences on ecosystems are increasing worldwide. Knowledge on the threshold levels of light pollution at which significant ecological impacts emerge and the size of dark refuges to maintain natural nocturnal processes is crucial to mitigate its negative consequences. Seabird fledglings are attracted by artificial lights when they leave their nest at night, causing high mortality. We used GPS data-loggers to track the flights of Cory’s shearwater *Calonectris diomedea* fledglings from nest-burrows to ground, and to evaluate the light pollution levels of overflown areas on Tenerife, Canary Islands, using nocturnal, high-resolution satellite imagery. Birds were grounded at locations closer than 16 km from colonies in their maiden flights, and 50% were rescued within a 3 km radius from the nest-site. Most birds left the nests in the first three hours after sunset. Rescue locations showed radiance values greater than colonies, and flight distance was positively related to light pollution levels. Breeding habitat alteration by light pollution was more severe for inland colonies. We provide scientific-based information to manage dark refuges facilitating that fledglings from inland colonies reach the sea successfully. We also offer methodological approaches useful for other critically threatened petrel species grounded by light pollution.

Artificial lights have provided important benefits to humanity, but they have also led to a loss of the natural nightscapes worldwide. More than two-thirds of the world human population lives under a light-polluted night sky, and the alteration of light levels at night hinders the visibility of the Milky Way by more than one-fifth^1^. Night sky degradation continues as light pollution levels increase at an annual rate of 6%^2^. Recently, light pollution has been recognised as an important threat to biodiversity conservation because it can cause cascading effects on ecosystem functioning in several ways^3^,^4^,^5^. Despite the numerous studies and reviews of the field, the underlying factors are far from being understood^5^, especially for secretive species. From a conservation point of view, mass mortality events of organisms are one of the most severe ecological consequences of light pollution, involving a wide range of taxa such as moths, sea turtles, passerine birds and seabirds^6^,^7^,^8^,^9^,^10^,^11^,^12^.

Petrels (Order Procellariiformes) are one of the most endangered groups of birds with commercial fisheries and introduced predators as their main threats^13^. They also suffer from mass mortality episodes caused by artificial lights. For a long time, it has been known that petrel fledglings are attracted and disorientated by artificial lights when they are leaving their nests for the first time and fly towards the sea^14^. More than 40 burrow-nesting petrel species, some of them critically endangered, are affected by lights (authors’ unpublished data). Every fledging season on islands where humans and petrels coexist, thousands of fledglings of different species are grounded by light pollution, a phenomenon called “fallout”^15^, being exposed to injuries or death by collision with human structures or vehicles, as well as predation^16^,^17^,^18^,^19^,^20^,^21^. To mitigate light-induced mortality, rescue campaigns are conducted by local governments and NGOs, releasing into the ocean a high proportion of the rescued birds. Around 10% of birds collected in the campaigns die, although light-induced mortality could be higher as lay people do not report on dead birds, thus 40% is a more accurate estimate^22^.

Because the study of petrels breeding on oceanic islands is challenging (they usually nest in underground burrows on remote, isolated and inaccessible areas, visit the colonies at night and spend most of their time at sea^23^), why petrels become disorientated by lights is far from being fully understood. The majority of our knowledge about the fallout comes from observational data from rescue campaigns, and mainly consists of the reporting of species identification, individual numbers, date and location^24^,^25^,^26^. Analyses of rescue campaign data have uncovered the main factors determining the number of grounded birds. Thus, more fledglings are grounded during moonless, windy and around peak fledging period nights^16^,^17^,^18^,^19^,^20^,^21^,^22^. Rescue campaign data have also described the spatial distribution of the fallout, indicating the most dangerous areas^27^,^28^,^29^. Positive correlations between the extent of urban areas and the number of grounded birds have emerged^17^,^19^. However, the highest numbers of grounded birds have not been reached in the most light-polluted areas according to satellite imagery^22^,^27^,^28^,^29^, probably because interaction with other factors, such as distribution of the breeding colonies and distance to artificial lights, play a crucial role. Unfortunately, rescue campaigns cannot identify the colony of origin of unbanded birds grounded by artificial lights, and an important information gap arises around how far birds are attracted to lights and which light intensity thresholds have a considerable effect^28^.

Current technological advances in remotely tracking systems of birds can help to understand why petrels are attracted to lights^29^,^30^. In this study, we have used affordable miniaturised GPS data loggers to track, for the first time, the flights of fledglings from their nest sites to ground. We describe flight characteristics to assess the extent and intensity of the impact of light pollution on the pathway of birds to the sea. Furthermore, using recovery rates of fledglings banded at colonies, we built generalised linear models to explain the contribution of geographical variables, such as colony elevation, distance to the coast and light pollution levels, on the light pollution impact on colonies. Knowledge of these factors has important implications for conservation and management; if only birds flying over or near light-polluted areas are susceptible to disorientation by lights, then light pollution would have a local effect. However, if birds from distant areas were grounded by lights, the consequences of light pollution would worsen by increasing its extent and impact, affecting a higher proportion of the island population. In this sense, Troy *et al.* proposed that fledglings could be attracted to lights from a long distance and concluded that a substantial part of the fallout corresponds to birds attracted back to land after reaching the sea^29^.

## Results

### Recovery of birds

We banded a total of 279 fledglings: 110 birds at natural nests in breeding colonies (first fliers) and 169 recovered fledglings from the rescue campaigns (second fliers). Of these, 63 first fliers and 94 second fliers were tagged with GPS data loggers. We retrieved 14 of 63 GPS-tracked first fliers during the rescue campaigns. Only five devices retained information. In the remaining nine, the GPS battery had expired before birds left their nests. One of 47 banded first fliers was recovered. We retrieved 14 of 94 GPS-tracked second fliers. In this case, all except one GPS contained information, as birds left their adopted nest-burrow right after sunset and no battery problems arose. Nine of 75 banded second fliers were recovered. Recovery rate was 14% both for first and second fliers (15 of 110 first fliers and 23 of 169 second fliers). Grounding rates of GPS-tagged and untagged birds released from one experimental site (second fliers) were similar, indicating that GPS attachment did not affect the probability of being grounded (14 recoveries of 94 tagged birds and nine recoveries of 69 untagged birds; Yates corrected *χ*^2^ = 0.011, *P* = 0.914).

### Flight characteristics

**Figure 1:**
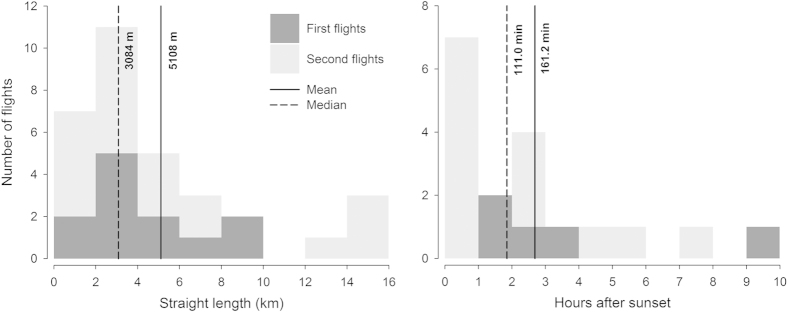
Histograms of distance from colony to rescue location and time of emergence after sunset.

**Figure 2:**
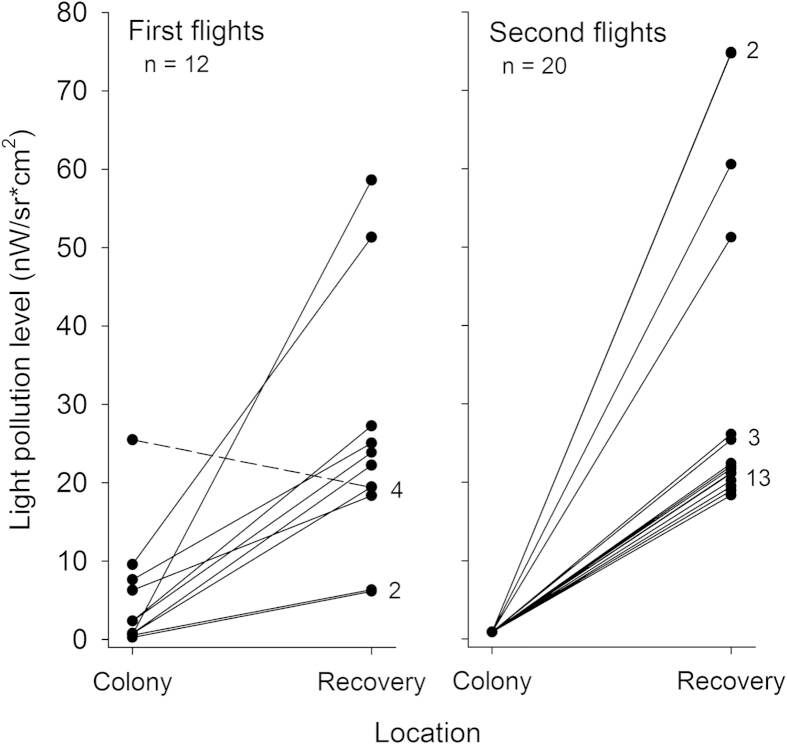
Light pollution levels at breeding colonies and at rescue locations. Numbers indicate the number of overlapped dots. A dashed line represents the only bird grounded in a less light-polluted area than its colony. Light pollution levels taken from a nocturnal satellite image produced by the Earth Observation Group, NOAA National Geophysical Data Center (see text).

**Figure 3:**
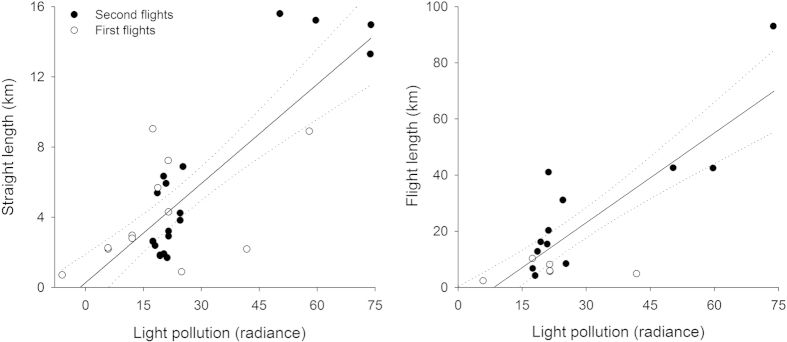
Relationships between flight distances and light pollution levels. Straight length is the distance between colonies and grounding locations in a straight line. Flight length is the cumulative distance flown by GPS-tracked shearwaters. Light pollution levels correspond to the difference in radiance values (nW/sr*cm^2^) of grounding locations minus colonies.

**Figure 4:**
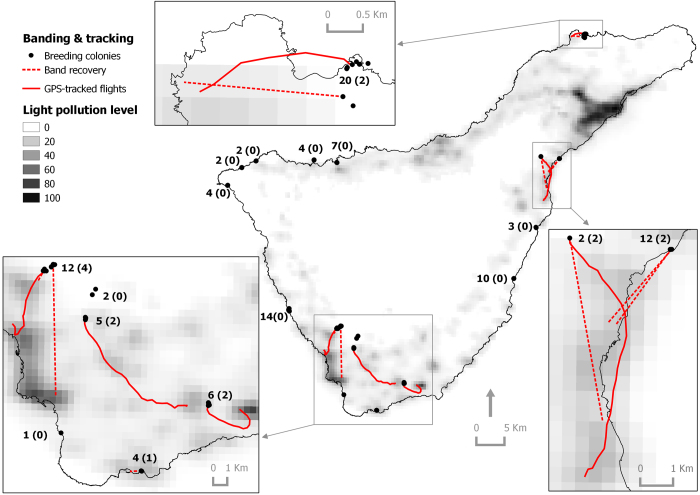
Map of Tenerife, Canary Islands, showing the banding locations at breeding colonies (dots), the straight lines linking nests and recovery locations (dashed lines) and GPS-tracked flights of Cory’s shearwaters (solid lines) of the natural approach, i.e. first flights (see details in main text). Light pollution levels correspond to radiance values (nW/sr*cm^2^) taken from a cloud-free composite of VIIRS night time lights corresponding to April and October of 2012 and produced by the Earth Observation Group, NOAA National Geophysical Data Center. Numbers indicate the number of fledglings banded at colonies and the number of birds recovered (in brackets). Map was created with QGIS version 2.0.1 (Open Source Geospatial Foundation Project, http://qgis.osgeo.org).

**Figure 5:**
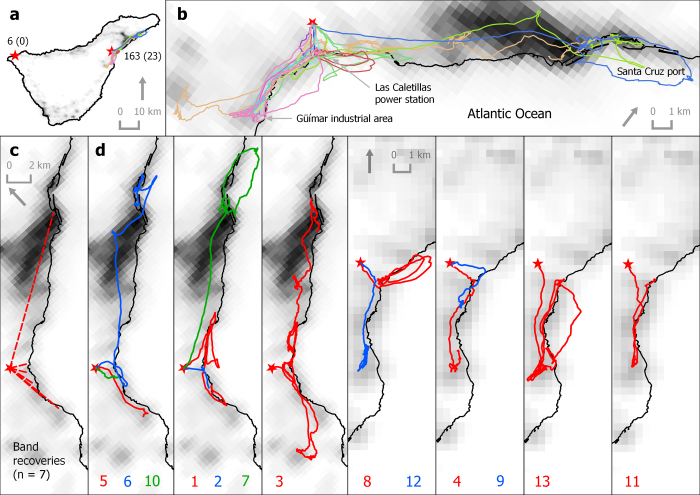
Map of Tenerife, Canary Islands, showing the release locations (**a**, stars), the critical illuminated areas (**b**), the straight lines linking nests and recovery locations (**c**, dashed lines), and GPS-tracked flights of Cory’s shearwaters (**d**, solid lines) of the experimental approach (see details in main text). Light pollution levels correspond to radiance values (nW/sr*cm^2^) taken from a cloud-free composite of VIIRS night time lights corresponding to April and October of 2012 and produced by the Earth Observation Group, NOAA National Geophysical Data Center. In a), numbers indicate the number of fledglings experimentally released and the number of birds recovered (in brackets). In **d**), numbers indicate the flight ID number used in our database. Map was created with QGIS version 2.0.1 (Open Source Geospatial Foundation Project, http://qgis.osgeo.org).

All rescued birds were found at lower elevations than their nesting colonies (or releasing site in the case of second fliers), and at locations less than 16 km from their colonies, with half of the birds being rescued within a radius of 3 km (Fig. 1 and SI 1). According to the VIIRS satellite imagery, birds were rescued in more light-polluted areas than their colonies, except one individual whose colony was located in an urban area with a pixel value of 25.5 nW/sr*cm^2^. The majority of birds were grounded in areas showing light pollution levels greater than 18 nW/sr*cm^2^(Fig. 2), and both straight length and cumulative length from nests to grounding locations and length of flights were positively related to light pollution levels (Fig. 3; Straight length: r = 0.470, n = 32, *P* = 0.004; and Flight length: r = 0.491, n = 18, *P* = 0.020). We recovered a lower proportion of birds from dark sky colonies or colonies with unpolluted-sky pathways to the sea (Figs. 4 and 5). Twelve of the 18 GPS-tracked birds flew over the sea; two from the first flight group and ten from the second flight group. The two first flights flew over the sea between headlands and lights were visible along the track (Fig. 4). Five second flights flew over the sea between headlands while following the coast (maximum distances to land lower than 250 m; Fig. 5). The remaining five second flights entered into the open sea moving off land 1-2.5 km (mean ± SD of maximum distances = 1593 ± 585 m, n = 5), and three landed on the water before grounding on land (Fig. 5).

Birds left nesting burrows 161.2 ± 153.8 (mean ± SD, n = 18) minutes after sunset on average (Fig. 1). After grounding, 13 of 18 GPS-tracked birds were rescued within 24 hours, two were rescued 24-48 hours after grounding, one was rescued four days after grounding and two birds were rescued five days after grounding.

Three of nine variables describing the flight characteristics were significantly different between first and second fliers (Table 1). Tortuosity of first flights was lower than second flights, while speed was greater among first flights. During first flights, birds lost altitude from the breeding colonies to light-polluted areas (cities). However, during second flights, birds were able to gain altitude, thus reducing their flight speed ( Figure SI 1).

### Factors affecting grounding rates

The proportion of grounded birds in relation to marked birds was positively related to the distance to the sea and the elevation of breeding colonies (Table 2). Models including explanatory variables of light pollution (i.e. mean and maximum light pollution levels within a radius of 3 km from the colony or the light pollution level at the colony) obtained higher AICc values and explained lower proportions of deviance, indicating worse fits.

## Discussion

### New insights on seabirds and artificial lights

Knowledge of the grounding risk of seabirds in relation to their exposure to light pollution on their colonies or their pathways to the sea is basic and fundamental for managing the seabirds and artificial lights problem^27^,^28^. Fledglings can be attracted to lights once they have successfully reached the sea^22^,^31^. It implies that fledglings raised in dark colonies, i.e. in areas unaltered by light pollution, can be affected by lights once they reach the sea and move along the coast, increasing the severity of this phenomenon and extending it to dark areas on the islands^28^,^29^. From a management perspective, unravelling the contribution of these birds to the numbers of the fallout is crucial. Our results indicate that their contribution does not seem to be substantial. First, we recovered a lower proportion of birds from dark sky colonies with unpolluted sky pathways towards the sea than in light-polluted colonies (Figs. 4 and 5). Second, all recovered birds were grounded at locations close to their breeding colonies, i.e. less than 16 km (but nine km if we only take into account first flights). No birds from the northern coast were found on the southern coast, or vice versa. Third, birds were grounded the same night when they left their burrows, i.e. there were no birds that reached the sea and were grounded by lights on subsequent nights. Thus, stranded birds are typically naïve birds that get locally disorientated on their maiden flight, and the contribution of fledglings attracted by lights back to the land from the ocean is not substantial to total fallout, at least on Tenerife Island, which contrasts what was reported for the Newell’s shearwater *Puffinus newellii* on Kauai, Hawaii^29^. We acknowledge important differences in intensity and distribution of light pollution between both islands which may explain these findings: Tenerife has an intense light pollution ring around its coast, whereas the darker Kauai shows dim polluted areas located in some coastal regions (maximum radiances = 103.6 and 16.5 nW/sr*cm^2^, respectively). Therefore, birds from inland colonies of Tenerife can view the lights before reaching the sea. In an intensely lit area (radiance = 27 nW/sr*cm^2^) located in the mainland Australia 15 km away from the nearest breeding colonies on Phillip Island, 237 short-tailed shearwaters *Ardenna tenuirostris* were found grounded during a single fledging season. This number is equivalent to one-quarter of the birds rescued on Phillip Island in the same year, and confirms that fledglings can be attracted to lights after reaching the ocean. However, its contribution to the general fallout is low, if we take into account the low light levels on the island and the huge breeding population size (maximum radiance = 5.3 nW/sr*cm^2^ and about 542,300 nests)^22^.

Our models indicate ‘distance to sea’ and ‘elevation’ are more important than light pollution level variables for explaining the vulnerability of colonies to artificial lights, providing information on so far untested hypotheses that inland colonies were more susceptible to lights than were coastal ones^18^,^22^. Thus, coastal breeding colonies are impacted less by light pollution, but artificial lights from urban areas act as a barrier for the movement of fledglings from inland colonies. Because of the high philopatry exhibited by petrels and the light-induced mortality of fledglings, one would expect a low recruitment rate at inland colonies, which may lead to their extinction in combination with other non-natural threats such as predation by introduced mammals or collision with power lines^17^,^21^. Adult petrels do not seem to be as affected by artificial lights as fledglings^16^,^17^,^18^,^19^. Breeders from inland colonies have to deal with artificial light barriers multiple times in their commuting flights during the breeding season. Information on how they manage artificial light barriers, e.g. using dark corridors or least-cost paths^32^, could help to understand why fledglings are grounded and propose measures based on scientific evidence for mitigating mortality of fledglings.

First flights were faster and straighter than second ones. During the first flights, birds seemed to be attracted to lights, whereas birds in their second flight seemed to be disorientated by lights (higher tortuosity values). These differences could be related to different nutritional statuses of first and second fliers, or fallout incident effects on second fliers, being released into a new nest or the flight experience acquired during the first flight. Explaining these findings deserves more investigation.

### Implications for conservation and management

An effort should be made to reduce light pollution to levels as low as possible in natural protected areas^33^, but also in adjacent areas, i.e. within 3 km from colonies, which should not show radiance levels > 10 nW/sr*cm^2^. According to our data, this action could reduce the attraction of shearwaters raised in protected areas to lit areas by 50%. However, the positive relationships between flight distances and light pollution levels suggest that the radius of light that attracts birds is dependent on light intensity (Fig. 3); greater light pollution intensities indicate larger attraction radii. Therefore, a 3 km buffer may not be enough to reduce attraction, especially in areas with highly polluted cities as noted in other studies^22^,^29^.

Furthermore, a majority of fledglings leave their nests during the first three hours after sunset^15, this study^, coinciding with the greatest usage of lights at night and, consequently, greatest light pollution levels^34^. To minimise conflict with the general public, an effort should be to inform residents and tourists about ecological and economic consequences of light pollution on native seabirds and to reduce the light emitted into the sky, especially during the fledging period. The touristic sector should be one of the main targets of awareness campaigns as it is one of the most contaminated with regard to light pollution. As an example, special certifications could be granted to resorts, restaurants, shops, and even golf courses showing environmental friendly activities in terms of energy efficiency, light emission and collaboration with rescue campaigns.

GPSs have also provided interesting information on the timing of rescue of birds, with some of them being rescued five days after being stranded. A key factor determining the mortality of fledglings grounded by light pollution is rescue date, with late rescued fledglings having a higher probability of death^35^. Thus, the rescue campaign design should be enhancement to avoid death by exhaustion, and birds should be released as soon as possible, or they should be provided with veterinary care, if required.

### Light-induced grounding rate and population size

A previous study estimated the proportion of fledglings grounded by lights in relation to the fledglings produced each year on Tenerife to be around 53%^19^, but the recovery rate of birds marked at their nests before fledging was 14%^this study^. We suggest the former to be an overestimation. Assuming a breeding success of 0.75 chicks per breeding pair^36^, 1,751 or 863 (the maximum and minimum numbers of rescued fledglings per year during our study period) grounded fledglings and an affection rate of 14%^this study^, the breeding population might range from 8,200 to 16,600 pairs. This figure is more than triple the 2,000-3,000 pairs estimated for Tenerife Island^37^ and warrants more detailed studies to assess the actual breeding population size.

### Methodological approaches

Knowledge of seabird attraction to light is limited mainly because of their secretive breeding behaviour within colonies^29^. This study constitutes a first attempt to track fledglings from their nests to places where they are grounded by lights. Novel technologies in animal tracking^30^ and affordable GPS data loggers (around 37 € per unit) allowed a research strategy in which we assumed many GPS units would be lost because birds would successfully reach the sea, or they would never be found by rescue campaigns. To give a sense of how risky our approach was, 52 Newell’s shearwater fledglings were banded at a colony on Kauai in a period of five years (1980-1985) and none were recovered in the rescue campaigns^17^. Based on a previously estimated grounding rate for Cory’s shearwaters on Tenerife, we expected to recover about 53% of the devices^19^.

Tracking first flights is challenging. Difficulties in the fieldwork, such as accessing breeding colonies at night and capturing the birds in their often-deep burrows made it a hazardous task. Uncertainty in predicting fledging date and battery lifespan made it a disappointing task: nine out of 14 recovered GPSs did not contain any information on flight tracks. Note that batteries expire rapidly if GPSs do not have satellite coverage, e.g. when birds are underground in their burrows. Tracking second flights was easier. Manipulation, GPS tagging and releasing at the adoption colony are conducted during daylight hours, thus facilitating fieldwork. Furthermore, birds leave the burrow on the subsequent nights, and the battery is full when the GPS is programmed to work. Despite potential differences arising because of different locations of their burrows (17 natural colonies *vs.* two places of releasing), we compared results from two approaches (natural first flights and post-recovery second flights) to evaluate grounding associated with light pollution. Both approaches are complementary, because first flights describe the actual problem whereas second flights help to identify critical illuminated areas, i.e. black spots where birds circle around lights (see, for example, Santa Cruz port, Güímar industrial area or Las Caletillas power station in Fig. 5b). Tracking of fledglings with devices able to download information remotely could provide a more complete picture of the seabird light attraction, reporting information of grounded birds and of those which have successfully reached the sea. Current inherent limitations of this technology make this approach challenging, but we foresee that the technological revolution will overcome these limitations in the near future^38^.

In our study, we have used the highest possible resolution nocturnal satellite imagery to assess light pollution levels, measured as radiance^39^. Other options have been employed for mapping the lights in the nightscape, such as aerial surveys of light emissions^40^,^41^ or models based on geographical features or position of lights^28^,^42^; however, these are too expensive and require complex analytical procedures. Despite the fact that satellite imagery could be too coarse for some isolated points of light; it constitutes a useful, easy and affordable tool for monitoring light pollution^27^, and those are key characteristics to be adopted by land policy makers^43^.

## Conclusions

The thresholds of light intensity and the size of ‘dark refuges’ needed to maintain natural ecosystem processes have been highlighted as future research topics on the ecological impacts of light pollution^44^. Our results shed some light on these two crucial topics for the attraction and disorientation problem seabirds face with artificial lights. A majority of birds were rescued at locations less than 10 km from their natal colonies and showing radiance values greater than 18 nW/sr*cm^2^. The implementation of measures taking into account these thresholds may curb the number of birds grounded by light pollution by 50%. Our methodological approaches are useful to study and mitigate light-induced mortality of other rare and threatened petrel species, but also to reveal secretive breeding aspects, such as commuting behaviour, colony locations and population sizes.

## Methods

### Study area and model species

Tenerife, the largest and one of the most mountainous Canary Islands (2,034 km^2^ and up to 3,718 m above sea level –a.s.l.), is located in the central part of the archipelago (27°37’–29°25’N, 13°20’–18°19’W). Approximately 900,000 people inhabit Tenerife permanently, the majority of whom are concentrated in towns and villages along the coastal slopes. In addition, 4.5 million tourists visit the island every year, and the hotel and self-catering bed supply currently exceeds 88,000^45^. The majority of these visitors (80%) are accommodated in large hotels and resorts on the southern part of the island^45^. Light pollution levels have increased during recent years in parallel with increased electricity consumption and population size^45^,^46^.

The Cory’s shearwater *Calonectris diomedea* is a medium-sized seabird (body mass, 600-800 g; wingspan, 112-126 cm), that breeds in Atlantic (Azores, Berlenga, Madeira, Selvagem and Canary Islands, *C. d. borealis* subspecies) and Mediterranean islands (*C. d. diomedea* subspecies). A single-egg clutch is laid in early June, and thus only up to one individual may fledge per nest and per year during late October to early November. It is the most abundant seabird species in the Canary Islands. On Tenerife, its breeding population has been estimated at 2,000-3,000 pairs (although this number could be a notable underestimation, see discussion), and it is widely distributed at elevations below 1,000 m a.s.l.^37^. As with other petrel species, the main causes of anthropogenic mortality include introduced predators, human harvest, fishery bycatch, and alteration of its breeding habitat^37^. In the Canary Islands, Azores, Madeira and among colonies in the Mediterranean archipelagos^21^,^24^,^27^, fledglings are grounded by artificial night lights. On Tenerife, an estimated 45-61% of fledglings are grounded by artificial light attraction (an average of 993 ± 217 fledglings are rescued annually^19^). A steady increase in the number of rescued individuals has been documented since 1990^26^.

### Rescue campaigns

Since 1990, the public has been requested to rescue stranded birds through awareness campaigns involving local media, seminars in primary and high schools and the distribution of posters, stickers and T-shirts. They are asked to collect and retain the birds, and then call ‘La Tahonilla’ wildlife rehabilitation centre, sponsored by the local government (Cabildo Insular de Tenerife), to inform of the rescue. Rescued birds are examined by ‘La Tahonilla’ staff for subsequent release into the sea. Injured birds are moved to the wildlife rehabilitation centre and held for rehabilitation, or they are euthanized. During rescue campaigns, about 6% of birds are found dead or had to be euthanized^19^.

### Field procedures

We used two field approaches to track the flights of shearwater fledglings from burrows to lit areas. For first flights (the natural approach), we visited breeding colonies distributed across the island at night between 20 October and 3 November of 2011-2014 (Fig. 4). Fledglings were captured by hand in the proximities of their nest-burrows when they came out the burrows to exercise their wings^23^. After manipulation, they were released back into their natural burrows.

A second approach using rescued birds also was implemented in 2012-2014. These individuals had initiated their maiden flights for the sea, but were grounded and rescued. These birds were released into burrows of two actual nesting-colonies (Fig. 5). We called these birds “second fliers”, as we aimed to track them during their second voyage from surrogate nest-sites. The breeding colonies where second fliers were born were unknown. All first and second fliers were banded with a unique metal ring and basic biometric measures were recorded at capture.

Field procedures were approved by the Ethics Committee for Animal Experimentation (Doñana Biological Station) in accordance with the Directive 2010/63/UE on animals used for scientific purposes. Regional and local governments (Gobierno de Canarias and Cabildo Insular de Tenerife) issued the permits for this study (file numbers: FYF 341/11, 132/12, 152/13, 162/14).

### GPS deployment

We employed CatTraQ (Catnip Technologies, USA) GPSs. The original case was removed, and the device was put in a heat-shrink tube for waterproofing. The final size was 27 × 55 × 12 mm and weighed 17 g, which represented <3% of the body mass of Cory’s shearwater fledglings. Devices were attached to the mid-dorsal feathers of birds with TESA tape^47^. GPSs were programmed to record a position every 30 seconds from approximately one hour after sunset. In 2013 and 2014, GPSs deployed to track the second flight of fledglings were programmed to record a position every 15 and 10 seconds, respectively.

### Environmental variables

We calculated duration (min), length (m), straight length (m), and speed (km/h) based on the information provided by GPS data loggers or ring recoveries. An index of tortuosity was calculated as 1-(straight length/length), which ranges from 0 (straight flight) to 1 (curved flight). Time lapsed (min) from sunset to the moment when the birds left the burrows was recorded by the ‘Timing’ variable. Apparent sunset times were obtained from the Earth Research System Lab, NOAA (available at http://www.esrl.noaa.gov/gmd/grad/solcalc/sunrise.html).

Light pollution levels of each location were taken from a cloud-free composite of VIIRS night-time lights corresponding to April and October of 2012, and were produced by the Earth Observation Group, National Oceanic and Atmospheric Administration (NOAA) National Geophysical Data Center (available at http://ngdc.noaa.gov/eog/viirs/download_monthly.html). VIIRS nocturnal imagery has a higher spatial resolution, a lower detection limit and no saturation^39^,^48^, which constitutes important advantages in relation to Defense Meteorological Satellite Program (DMSP) imagery previously used in other seabird studies^26^,^27^,^28^,^29^. For GPS-tracked flights, we calculated the mean and maximum light pollution levels by intersecting flight tracks with the nocturnal image. For colonies, the pixel value of the satellite image intersected with the centre of the colony was used as a measure of exposure to light pollution. Mean and maximum light pollution levels were calculated for an arbitrarily selected 3 km radius from the centre colony. Burrows more than 1 km away were assigned to different colonies (average distance between the nearest burrows was 80 m).

A Digital Elevation Model (DEM) with a cell size of 25 × 25 m and an accuracy of 1 m of horizontal and vertical resolution respectively was obtained from the Digital Atlas of Tenerife^49^ and used to calculate elevation of every GPS location, as well as distance to the sea and elevation of colonies. Geographical analyses were conducted in QGIS version 2.0.1 (Open Source Geospatial Foundation Project, http://qgis.osgeo.org). Flight altitude was obtained from GPS data loggers output.

### Statistical analysis

We used randomisation tests (without replacement) to compare characteristics of first and second flights (Table 1). Because of the small sample sizes, we calculated *P*-values by running 8,568 iterations for information obtained from GPSs (8,568 different permutations for a sample of 18 and a group size of 13). For the remaining variables with a larger sample size (Table 1), 9,999 iterations were used to calculate *P*-values (225,792,840 different permutations for a sample of 32 and a group size of 20). To test potential relationships between flight distances and light pollution levels, Spearman’s tests on independence were used. The null distributions of statistics tests were one-tailed and approximated via Monte Carlo resampling (9,999 replications).

To model the proportions of grounded birds, we used a GLM with a two-column object for response (rescued/non-rescued birds), binomial error distribution and logit link^50^. We used two physical explanatory variables: colony elevation and distance from the colony to the sea; and three light pollution metrics: exposure at colony location (single pixel value) and mean and maximum values in a 3 km radius from the colony. Because collinearity among these variables was high (variance inflated factors ranged from 1.7 to 9.7), and to avoid over-parameterization, we built univariate models, i.e. including only one independent variable per model. Corrected Akaike Information Criterion (AICc) was employed to select the best models. The lower the AICc, the better the model^51^.

Statistical analyses were conducted in R version 3.0.3 (R Foundation for Statistical Computing, Vienna, Austria), using the MASS, car and coin packages.

## Additional Information

**How to cite this article**: Rodríguez, A. *et al.* GPS tracking for mapping seabird mortality induced by light pollution. *Sci. Rep.* **5**, 10670; doi: 10.1038/srep10670 (2015).

## Supplementary Material

Supporting Information

## Figures and Tables

**Table 1:** Flight details of Cory’s shearwater fledglings grounded by artificial lights on Tenerife, Canary Islands.

**Variables**	**First flights**		**Second flights**		*P***-value**
	**Mean ± SD**	**range**	**Mean ± SD**	**range**	
**Timing** (min)	224.52 ± 192.32	100.20–560.90	136.86 ± 137.28	34.20–479.60	0.297
**Duration** (min)	8.78 ± 4.66	3.20–16.10	93.50 ± 110.65	8.90–340.83	0.071
**Length** (m)	6325.8 ± 3054.2	2337–10296	26151.5 ± 24625.2	4195–93044	0.074
Straight length (m)	4092.2 ± 2943.6	706–9038	5717.6 ± 4921.3	1690–15597	0.307
**Tortuosity**	0.226 ± 0.200	0.064–0.554	0.664 ± 0.229	0.184–0.959	0.002**
**Speed**^a^ (km/h)	44.79 ± 12.55	35.59–66.60	29.61 ± 4.93	19.02–37.85	<0.001***
Light pollution levels					
**max. level**	31.79 ± 18.96	6.13–51.33	41.43 ± 32.34	18.93–103.64	0.542
**mean level**	14.68 ± 8.75	2.18–26.19	18.71 ± 9.52	10.01–44.67	0.444
** **colony/grounding place ratio	0.241 ± 0.359	0.011–1.313	0.037 ± 0.012	0.012–0.049	<0.001***

Variables in bold were calculated from information provided by GPS data loggers (sample sizes: n = 5 for first flights, n = 13 for second flights). The sample size for the remainder variables is 12 and 20 for first and second flights, respectively (total sample equals 32). *** and ** indicate *P*-values <0.001 and <0.01, respectively. ^a^ until birds rest on the sea surface.

**Table 2:** Results of the candidate models explaining the grounding rate of breeding colonies to artificial lights.

**Explanatory variables**	**Estimate ± SE**	**95% CI**	**AICc**	**ΔAICc**	**% deviance explained**
Distance to sea	0.0004 ± 0.0001	0.0001 , 0.0006	39.29	0.00	30.7
Elevation	0.0036 ± 0.0012	0.0011 , 0.0060	40.00	0.71	28.3
Mean level of light pollution	0.2456 ± 0.1028	0.0455 , 0.4551	42.65	3.36	19.4
Maximum level of light pollution	0.0333 ± 0.0144	−0.0047 , 0.0622	43.26	3.97	17.4
Exposure to light pollution	0.0557 ± 0.0418	−0.0341 , 0.1365	46.80	7.52	5.4
Null	—	—	45.82	6.53	0.0

We employed the proportion of rescued birds for each colony as a variable response (see text for details).
