# Supplementary material for: GPS tracking for mapping seabird mortality induced by light pollution
Source: Sci Rep. 2015 Jun 2;5:10670. doi: 10.1038/srep10670 (PMC4451840; doi:10.1038/srep10670)
Supplement: Supporting Information [file srep10670-s1.pdf]

Supporting Information

Figure SI 1

GPS tracking for mapping seabird mortality induced by light pollution

Airam Rodríguez[1,2,3*], Beneharo Rodríguez[3], and Juan J. Negro[1]

[1] Department of Evolutionary Ecology, Estación Biológica de Doñana (CSIC), Avda. Américo Vespucio s/n, 41092 Seville, Spain

[2] Research Department, Phillip Island Nature Parks, P.O. BOX 97, 3922 Cowes, Victoria, Australia

[3] Canary Islands' Ornithology and Natural History Group (GOHNIC), La Malecita s/n, 38480 Buenavista del Norte, Tenerife, Canary Islands, Spain

* Author for correspondence: Airam Rodríguez
Address: Research Department, Phillip Island Nature Parks, P.O. BOX 97, 3922 Cowes, Victoria, Australia
Phone: + 61 (3) 59512800
E-mail: airamrguez@ebd.csic.es

Figure SI 1 Profiles of Cory's shearwater flights from their colonies to rescue locations. Flight altitude (grey bars), elevation (dark grey) and light pollution levels (black lines) are shown. Information was obtained from GPS data loggers, a digital elevation model and a nocturnal satellite image, respectively (see text for details). a) and b) indicate first and second flights, respectively. Numbers in bold indicate the flight number used in our database.

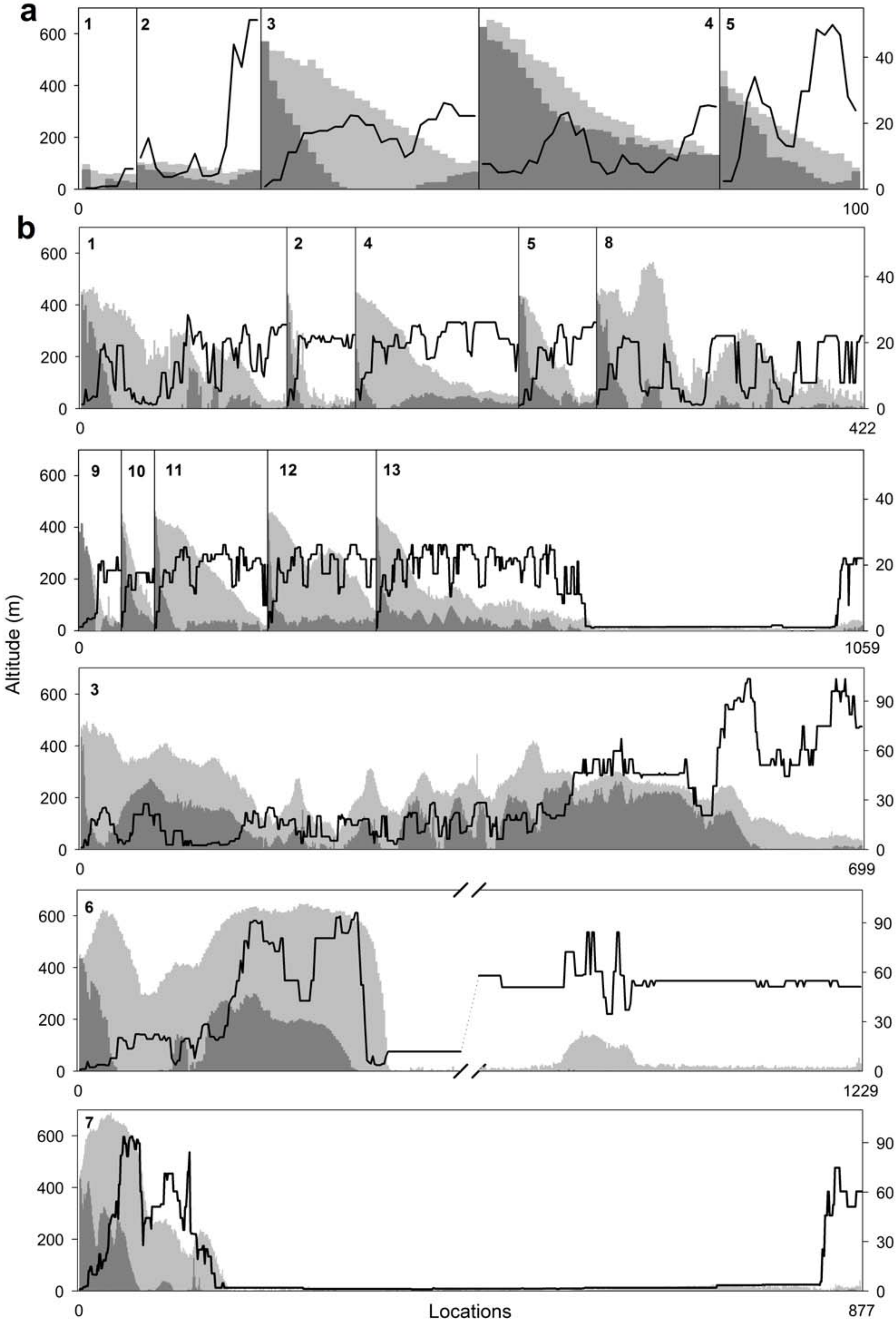
a
b
Altitude (m)
Light pollution level (nW/sr*cm2)
Locations
1
2
3
4
5
8
9
10
11
12
13
6
7
0
100
422
1059
699
1229
877
